# High prevalence of multidrug-resistant *Enterobacterales* carrying extended-spectrum beta-lactamase and AmpC genes isolated from neonatal sepsis in Ahvaz, Iran

**DOI:** 10.1186/s12866-024-03285-6

**Published:** 2024-04-24

**Authors:** Sima Mansouri, Mohammad Savari, Arash Malakian, Effat Abbasi Montazeri

**Affiliations:** 1 Infectious and Tropical Diseases Research Center, Health Research Institute, Ahvaz Jundishapur University of Medical Sciences, Ahvaz, Iran; 2 Department of Microbiology, Faculty of Medicine, Ahvaz Jundishapur University of Medical Sciences, Ahvaz, Iran; 3 Department of Pediatrics, Imam Khomeini Hospital, Ahvaz Jundishapur University of Medical Sciences, Ahvaz, Iran

**Keywords:** Neonatal sepsis, *Enterobacterales*, Multidrug resistant, AmpC, ESBL, ERIC-PCR

## Abstract

**Objectives:**

In the recent years, multidrug resistant (MDR) neonatal septicemia-causing *Enterobacterales* has been dramatically increased due to the extended-spectrum beta-lactamases (ESBLs) and AmpC enzymes. This study aimed to assess the antibiotic resistance pattern, prevalence of ESBLs/AmpC beta-lactamase genes, and Enterobacterial Repetitive Intergenic Consensus Polymerase Chain Reaction (ERIC-PCR) fingerprints in *Enterobacterales* isolated from neonatal sepsis.

**Results:**

In total, 59 *Enterobacterales* isolates including 41 (69.5%) *Enterobacter* species, 15 (25.4%) *Klebsiella pneumoniae* and 3 (5.1%) *Escherichia coli* were isolated respectively. Resistance to ceftazidime and cefotaxime was seen in all of isolates. Furthermore, all of them were multidrug-resistant (resistant to three different antibiotic categories). The phenotypic tests showed that 100% of isolates were ESBL-positive. Moreover, AmpC production was observed in 84.7% (*n* = 50/59) of isolates. Among 59 ESBL-positive isolates, the highest percentage belonged to *bla*_CTX−M−15_ gene (66.1%) followed by *bla*_CTX−M_ (45.8%), *bla*_CTX−M−14_ (30.5%), *bla*_SHV_ (28.8%), and *bla*_TEM_ (13.6%). The frequency of *bla*_DHA_, *bla*_EBC_, *bla*_MOX_ and *bla*_CIT_ genes were 24%, 24%, 4%, and 2% respectively. ERIC-PCR analysis revealed that *Enterobacterales* isolates were genetically diverse. The remarkable prevalence of MDR *Enterobacterales* isolates carrying ESBL and AmpC beta-lactamase genes emphasizes that efficient surveillance measures are essential to avoid the more expansion of drug resistance amongst isolates.

**Supplementary Information:**

The online version contains supplementary material available at 10.1186/s12866-024-03285-6.

## Introduction

Estimates indicate that neonatal sepsis affects 2,202 out of 100,000 live births globally, with mortality rates varying from 11 to 19% [1]. The Global Sepsis Alliance states that infections leading to sepsis are responsible for roughly one-fifth of the 2.7 million neonatal deaths that occur worldwide every year [1]. Neonatal sepsis is known as one of the most significant reasons for confinement of newborns in neonatal intensive care units (NICUs) [2, 3]. It is classified either as an early-onset neonatal sepsis (EOS) or as late-onset sepsis (LOS). EOS is explained as sepsis occurring in newborns less than 3 days old, and LOS is defined as sepsis occurring in infants aged 4 to 90 days [4]. There are various bacteria causing neonatal sepsis globally. Gram negative bacteria isolated from neonatal sepsis have been reported more than Gram positive bacteria in developing countries [5]. Today, catastrophic emergence of neonatal sepsis along with antimicrobial resistance to widely used antibiotics is a great challenge [6].

In the last few years, resistance to broad-spectrum beta-lactam antibiotics in members of the *Enterobacterales* family has been dramatically increased due to the production of extended-spectrum beta-lactamases (ESBLs) and/or AmpC enzymes [7]. ESBL genes are carried to other bacteria via bacterial plasmids. CTX-M, TEM and SHV beta-lactamases are the most prevalent ESBLs identified in *Enterobacterales* strains [8].

In some species of *Enterobacterales*, AmpCs enzymes can either encoded by chromosomal genes (cAmpCs) or can be found as acquired plasmid-mediated enzymes (pAmpCs). There are divers lineages of pAmpC genes, originating from cAmpC genes harbored by different Gram negative species which can be put into five phylogenetic classes, including *Enterobacter* class (MIR, ACT), the *Citrobacter freundii* class (CMY-2-like, LAT, CFE), the *Morganella morganii* class (DHA), the *Hafnia alvei* class (ACC), and the *Aeromonas* class (CMY-1-like, FOX, MOX) [9].

Overall, in clinical settings, AmpC and ESBLs producing gram-negative pathogens cause many health problems, including treatment failure, increased hospitalization time and treatment costs, and ultimately increased mortality [10]. Another important problem in the clinical field is that AmpC carriers are considered as a hidden warehouse for ESBLs and cause problems in their detection. Therefore, the simultaneous existence of these enzymes makes the treatments face a serious problem [11].

For evaluation of the clonality and genetic diversity of *Enterobacterales*, several methods are used. Based on evidence in the essays, Polymerase Chain Reaction (PCR)-based typing methods are the most popular owning to the high speed to gain results [12]. Enterobacterial Repetitive Intergenic Consensus (ERIC) sequences are repetitive imperfect palindromes, 127 bp in size which occur in multiple copies on bacterial genomes [13]. To compare clusters generated, ERIC-PCR is employed because it is affordable, simple to perform and a swift method [14]. This work aimed to assess the antibiotic resistance pattern, prevalence of ESBLs/AmpC beta-lactamase genes and ERIC-PCR fingerprints in *Enterobacterales* isolated from neonatal sepsis.

## Materials and methods

### Ethics statement

This study was approved by the Ethics Committee of Ahvaz Jundishapur University of Medical Sciences, Ahvaz, Iran (IR.AJUMS.MEDICINE.REC.1398.046). Informed written consent form was obtained from parents or legal guardians of any participant under the age of 16.

### Study design and sampling

This study was carried out in the span of 18 months (May 2019 to October 2020) and involved 850 neonates with clinical symptom of sepsis (fever, poor feeding, respiratory distress, hypothermia, gastrointestinal and/ or central nervous system symptoms) hospitalized in NICU of Imam Khomeini and Abuzar hospitals related to the Ahvaz Jundishapur University of Medical Sciences in Ahvaz city, Iran. Blood collection was done with aseptic precautions before beginning of antibiotics use and to each of two bottles containing of trypticase soy broth (TSB), 1–2 ml of blood was added (BaharafshanCo., Tehran, Iran). Incubation of both bottles was done in aerobic condition at 37 °C for 7 days. According to the available and accepted evidence by the international community, the diagnosis of sepsis was considered based on the positive culture result [15].

### Microbial identification

Sheep blood agar and MacConkey agar (Merck Co., Darmstadt, Germany) were used to do subculture after 24–48 h of incubation. The same instruction was repeated until the 7th day before blood cultures were regarded as negative of bacterial growth. Growth of potential pathogens in blood culture is always significant, even if it grows in a single vial. Even coagulase-negative staphylococci that grow in a single bottle during the neonatal period were considered to be infectious agents. Therefore, the growth of potential pathogens in a single vial and in pure culture was considered as significant growth. Also, if both broths contained the same organism, the growth was recognized as pathogenic. Standard biochemical tests were applied for identification of the *Enterobacterales* isolates [8]. To store some of the pure colonies for a long time, they were suspended in 15% glycerol-TSB (Sigma-Aldrich, St. Louis, MO, USA) and then put in − 80 °C.

### Antimicrobial susceptibility testing (AST)

All *Enterobacterales* isolates were evaluated for antimicrobial susceptibility by disc diffusion method in accordance with the Clinical and Laboratory Standards Institute (CLSI) recommendation [16]. The antibiotics used for susceptibility testing were: ampicillin (10 µg), ampicillin/sulbactam (10/10 µg), piperacillin/tazobactam (100/10 µg), cefoxitin (30 µg), ceftazidime (30 µg), cefotaxime (30 µg), imipenem (10 µg), meropenem (10 µg), amikacin (30 µg), gentamicin (10 µg), ciprofloxacin (5 µg), cotrimoxazole (25 µg) (MAST, Berkshire, UK). Multidrug resistance (MDR) were described based on nonsusceptibility to at least one agent in three or more antimicrobial categories [17]. *Escherichia coli* ATCC 25,922 and *Pseudomonas aeruginosa* ATCC 27,853 were used as strains for quality control.

### Phenotypic detection of ESBLs and AmpC

The *Enterobacterales* isolates with resistance to one or more of third-generation cephalosporins were investigated for confirmation of ESBL production by double-disc synergy test (DDST), according to CLSI procedure [16]. The test was conducted employing ceftazidime (30 µg) and cefotaxime (30 µg) disks separately and each of them in combination with clavulanic acid (10 µg) disk (Mast group, Merseyside, UK). Enhancement of inhibition zone size of ≥ 5 mm in the existence of clavulanic acid was a sign of ESBL positive isolates [16]. For the positive ESBL control, *Klebsiella pneumoniae* ATCC 700,603 and for negative ESBL control, *Escherichia coli* ATCC 25,922 were utilized. Furthermore, to test the *Enterobacterales* isolates for AmpC beta-lactamase production, cefoxitin disc (30 µg) was used [18]. Isolates having zone diameters of less than 18 mm were regarded potential AmpC beta-lactamase producers [18].

### PCR detection of ESBLs and AmpC genes

The boiling method was used to extract total DNA as described previously [17]. Nano Drop Spectrophotometer PROMO (Thermo Scientific, USA) and agarose gel electrophoresis respectively were used to evaluate the DNA quantity and quality. Based on the studies mentioned formerly, the identification of ESBLs (*bla*_CTX−M_, *bla*_CTX−M−14_, *bla*_CTX−M−15_, *bla*_TEM_ and *bla*_SHV_) and AmpC genes (*bla*_CIT_, *bla*_MOX_, *bla*_DHA_ and *bla*_EBC_) was performed by PCR in the BIO-RAD C1000 thermal cycler (Applied Biosystems, USA) [17, 19]. Table 1 showed the sequences of all primers. The final volume of PCR was 25 µl containing 12.5 µl of PCR master mix (Sinaclon, Tehran, Iran), 1 µl of each primer (10 pmol), 5 µl of DNA sample, and 5.5 µl of nuclease-free water. Analysis of the amplicons was done by agarose gel electrophoresis stained with safe stain (Sinaclon Co., Tehran, Iran). The amplicons were visualized using an ultraviolet gel documentation device (Protein Simple, San Jose, CA, USA).

**Table 1:** The primer sequences used in this study

Target gene	Oligonucleotide sequence (5′ to 3′)	Annealing temperature (◦C)	Product size (bp)	Reference
*bla*_TEM_	F: GAGTATTCAACATTTCCGTGTC R: TAATCAGTGAGGCACCTATCTC	60	800	17
*bla*_SHV_	F: CGCCTGTGTATTATCTCCCTGTTAGCC	60	843	17
	R: TTGCCAGTGCTCGATCAGCG			
*bla*_CTX−M−14_	F: TTATGCGCAGACGAGTGCGGTG	55	120	19
	R: TCACCGCGATAAAGCACCTGCG			
*bla*_CTX−M−15_	F: GAGCCGACGTTAAACACCGCCA R: GCTGCACCGGTGGTATTGCCTT	58	156	19
*bla*_CIT_	F: TGGCCAGAACTGACAGGCAAA R: TTTCTCCTGAACGTGGCTGGC	57	462	17
*bla*_MOX_	F: GCTGCTCAAGGAGCACAGGAT R: CACATTGACATAGGTGTGGTGC	57	520	17
*bla*_DHA_	F: AACTTTCACAGGTGTGCTGGGT	57	405	17
	R: CCGTACGCATACTGGCTTTGC			
*bla*_EBC_	F: TCGGTAAAGCCGATGTTGCGG R: CTTCCACTGCGGCTGCCAGTT	57	302 Variable	17
ERIC	F: ATGTAAGCTCCTGGGGATTCAC R: AAGTAAGTGACTGGGGTGAGCG	51		11

### ERIC- PCR

Primers described previously were applied to perform ERIC-PCR on *Enterobacterales* isolates [11]. Each PCR reaction (15 µl) contained 7.5 µl of master mix, forward and reverse primers (10 pmol) each 0.6 µl, template DNA 3 µl, and distilled water up to 15 µl. Following temperatures were used for ERIC-PCR: initial denaturation of 94 ◦C for 3 min and 35 cycles of 94 ◦C for 1 min, 51 ◦C for 1 min and 72 ◦C for 1 min followed by a final extension of 72 ◦C for 7 min. The electrophoresis of amplicons was carried out on 2% agarose gel with DNA safe stain along with a 100 bp DNA ladder. The gel electrophoresis images were assessed by BioNumerics 6.6 version software (Applied Maths; NV Keistraat, Sint-Martens-Latem, Belgium). For estimation of the similarity of ERIC-PCR patterns, Dice coefficients were used. For construction of dendrograms, the unweighted pair group method with arithmetic averages (UPGMA) was used and isolates were classified into the same cluster with a cutoff value of 80% similarity.

### Statistical analysis

For statistical analysis, SPSS version 23.0 (Armonk, NY, USA) was used. The variables were indicated as the descriptive frequencies and the data were assessed by Fisher’s exact test. If *P*-value was less than 0.05, it was interpreted as statistically significant difference.

## Results

### Frequency of *Enterobacterales* isolates

Among 850 neonates suspected of having sepsis, 320 of them had positive blood culture (Gram positive, *n* = 216; Gram negative, *n* = 104). Among these 320 positive samples, *Enterobacterales* isolates were detected in 59 samples. The most common *Enterobacterales* isolates were *Enterobacter* species (69.5%, *n* = 41/59), followed by *K. pneumoniae* (25.4%, *n* = 15/59), and *E. coli* (5.1%, *n* = 3/59). Characteristics of 59 *Enterobacterales* isolates have shown in supplementary files 1.

### Resistance rates of bacteria to antibiotics

**Fig. 1:**
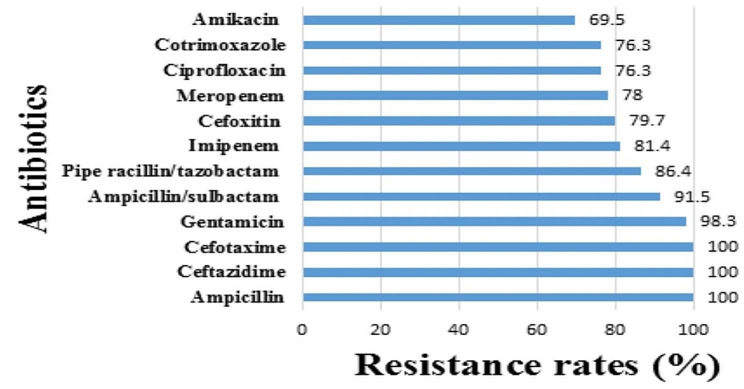
Antibiotic resistance rates of 59 *Enterobacterales* isolates

All 59 isolates were MDR. In total, 20 (33.8%) of them were resistant to all antibiotics (supplementary files 1). All tested isolates (100.0%) were resistant to ampicillin, ceftazidime, and cefotaxime. Distribution of resistance to other antibiotics was as follows: gentamicin (98.3%), ampicillin/sulbactam (91.5%), piperacillin/tazobactam (86.4%), imipenem (81.4%), cefoxitin (79.7%), meropenem (78.0%), ciprofloxacin (76.3%), cotrimoxazole (76.3%), and amikacin (69.5%) (Fig. 1).

### Phenotypic detection of ESBLs and AmpC

**Fig. 2:**
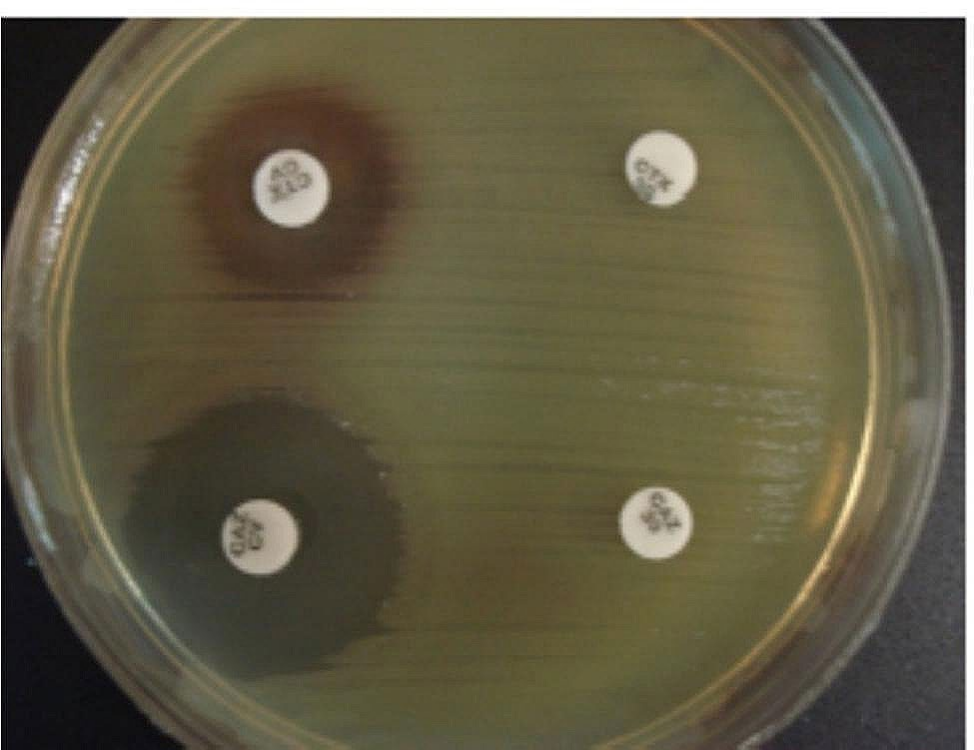
Confirmation of extended-spectrum beta-lactamase (ESBL) production by double-disc synergy test (DDST)

According to the results of DDST, 100% of the isolates were ESBL positive. Also, 50 (84.7%) isolates showed an inhibition zone ≤ 18 mm against cefoxitin (30 µg). The phenotypic detection of ESBLs was shown in Fig. 2.

### PCR detection of ESBLs and AmpC genes

**Fig. 3:**
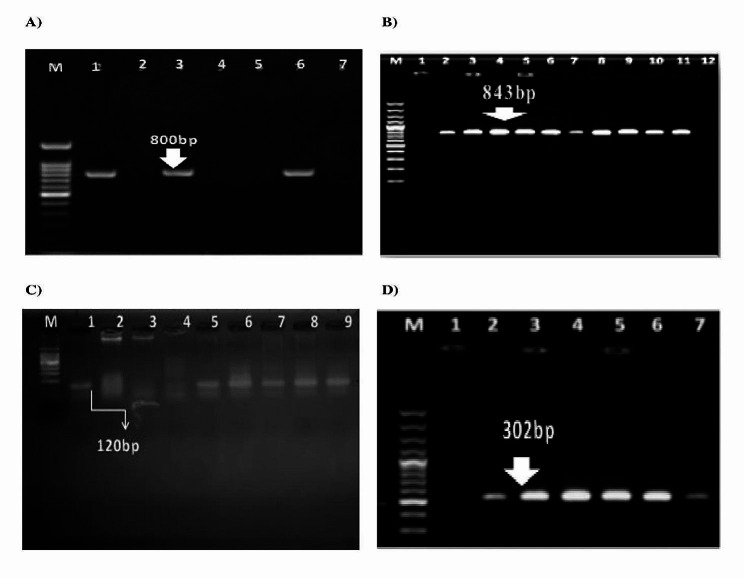
**A**) Simplex PCR for *bla*_TEM_ gene (800 bp); M: DNA ladder (100 bp); Lanes 1: positive control; Lane 2: negative control: DNA/RNA free water; Lanes 3 and 6: *Enterobacter* isolates positive for *bla*_TEM_ gene; Lanes 2,4,5, and 7: isolates negative for *bla*_TEM_ gene. **B**) Simplex PCR for *bla*_SHV_ gene (843 bp); M: DNA ladder (100 bp); Lanes 1: negative control: DNA/RNA free water; Lane 2: positive control; Lanes 3 to 11: *Enterobacter* isolates positive for *bla*_SHV_ gene; Lanes 12: *Enterobacter* isolate negative for *bla*_SHV_ gene. **C**) Simplex PCR for *bla*_CTX−M−14_ gene (120 bp); M: DNA ladder (100 bp); Lanes 1: positive control; Lane 2: negative control: DNA/RNA free water; Lanes 3 and 4: *Enterobacter* isolates negative for *bla*_CTX−M−14_ gene; Lanes 5 to 9: isolates positive for *bla*_CTX−M−14_ gene. **D**) Simplex PCR for *bla*_EBC_ gene (302 bp); M: DNA ladder (100 bp); Lanes 1: negative control: DNA/RNA free water; Lane 2: positive control; Lanes 3 to 7: *Enterobacter* isolates positive for *bla*_EBC_ gene

All phenotypic ESBL-producing *Enterobacterales* isolates were positive for at least one ESBL genes. The frequency of ESBL genes was as follows: *bla*_CTX−M−15_ (66.1%), *bla*_CTX−M_ (45.8%), *bla*_CTX−M−14_ (30.5%), *bla*_SHV_ (28.8%), and *bla*_TEM_ (13.6%). In total, 37.3% (*n* = 22/59) of isolates were AmpC positive by PCR method. Moreover, results of PCR screening for the existence of AmpC genes showed that 12 (24%), 12 (24%), 2 (4%), and 1 (2%) of *Enterobacterales* isolates contained *bla*_DHA,_*bla*_EBC_, *bla*_MOX_, and *bla*_CIT_ genes, respectively (supplementary files 1). The PCR products of some ESBLs and AmpC genes are shown in Fig. 3. According to the Fisher’s exact test, there was no significant difference between phenotypic test and PCR in the detection of ESBLs (*P*-value > 0.999) (Table 2). However, there was a significant difference between phenotypic test and PCR in the detection of AmpC (*P*-value = < 0.00001) (Table 2).

**Table 2:** Detection of extended spectrum β-lactamase (ESBLs) and AmpC by phenotypic test and polymerase chain reaction (PCR)

	Phenotypic test		PCR		P-value
**ESBLs**	Positive n (%)	Negative n (%)	Positive n (%)	Negative n (%)	
	59 (100.0%)	0 (0.0%)	59 (100.0%)	0 (0.0%)	> 0.009
**ApmC**	50 (84.7%)	9 (15.3%)	22 (37.3%)	37 (63.7%)	< 0.00001

### ERIC- PCR analysis

**Fig. 4:**
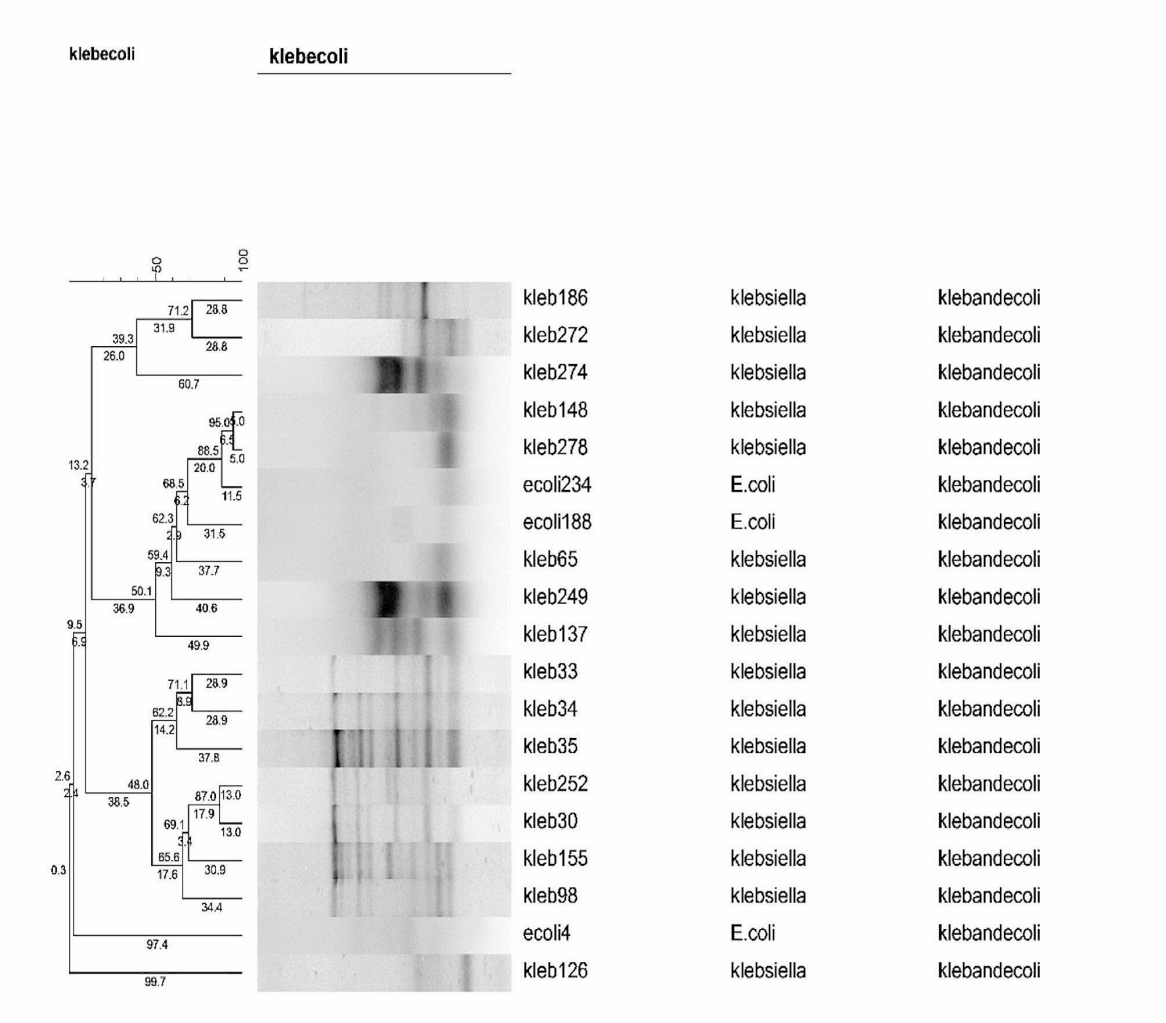
Dendogram of ERIC-PCR results for *Klebsiella pneumoniae* and *Escherichia coli* isolates

**Fig. 5:**
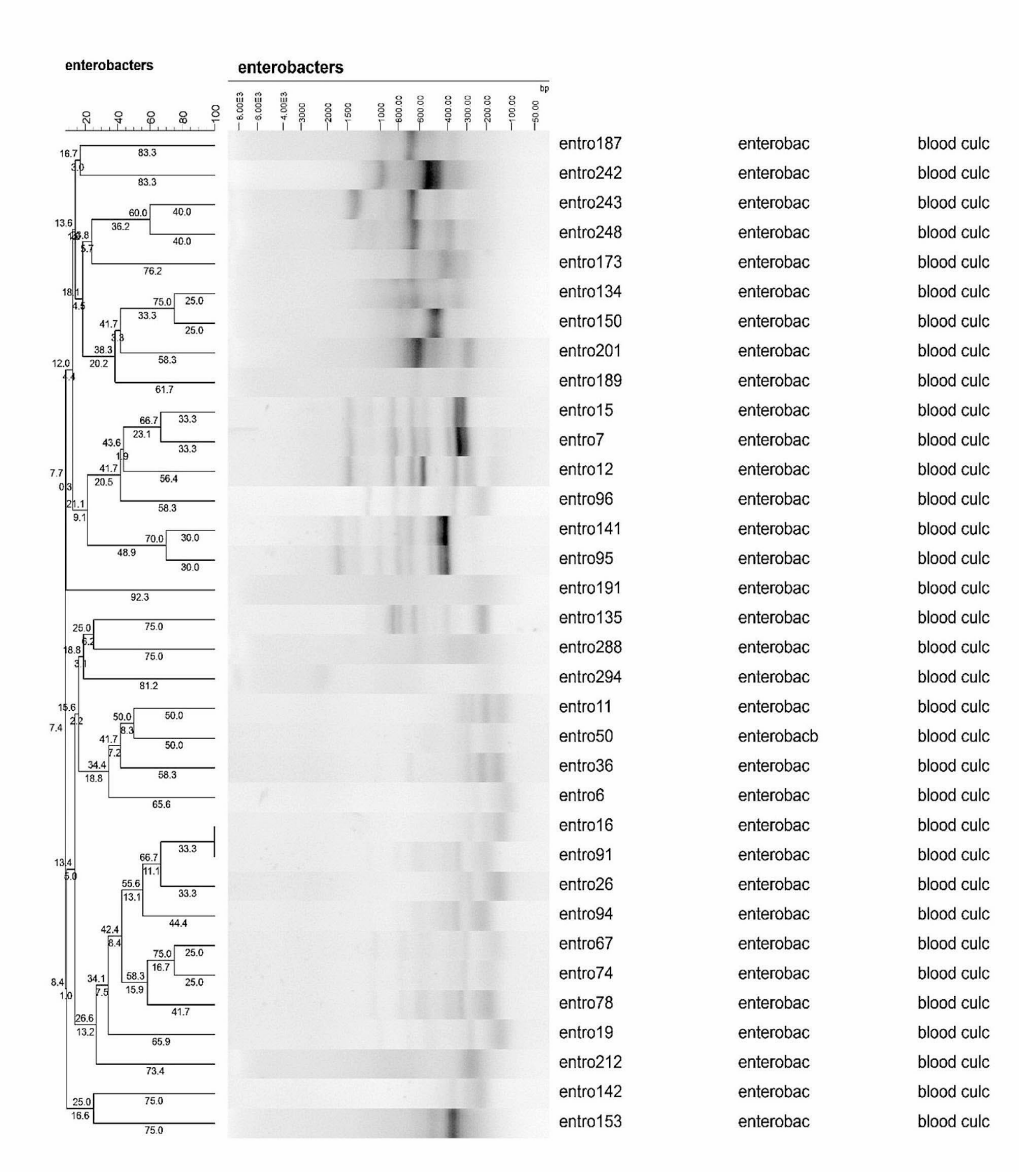
Dendogram of ERIC-PCR results for *Enterobacter* isolates

There was a high diversity among *Enterobacterales* isolates according to the ERIC-PCR analysis. The *K. pneumoniae* and *E. coli* isolates were divided into 16 clusters, while two of them were multitone and 14 were singleton (Fig. 4). The *Enterobacter* isolates were also categorized as 33 clusters. There was only one multitone and 32 singletons (Fig. 5).

## Discussion

Neonatal sepsis is still considered as one of the main health issues in the world [4]. In this study, the incidence of neonatal sepsis was confirmed by blood culture to be 37.6% (320/850). Prevalence of sepsis is diverse in different parts of Iran (4.14–46.49%) [4] and other countries including India (58%) [18] and Ethiopia (39.5%) [19]. The reasons for these geographical differences are the different preventive strategies of each country, different clinical features for the diagnosis of sepsis, varying sensitivity and specificity of culture methods between laboratories, the health status of mothers during pregnancy, and the socio-economic conditions of the countries [4]. It was in 2007 that the Iranian Nosocomial Infection Surveillance System (INIS) was created [20]. However, it is important to acknowledge the limitations of this system, which encompass both under-reporting and over-reporting [20]. Ensuring the reliability of information necessitates the activation of Infection Control Link Nurses (ICLN), the empowerment of Infection Control Nurses (ICNs) through specialized training, and clarification of job descriptions [20].

In this study, *Enterobacterales* isolates were found in 59 out of 320 positive samples (18.4%), which was lower than in previous studies [8, 21]. The size of the studied sample, the diagnostic methods used to identify bacteria, and the geographical area studied are among the effective factors in creating these differences.

In this study, *Enterobacter* species (69.5%) were the most prevalent *Enterobacterales* isolates responsible for causing neonatal sepsis. In line with this finding, a previous study by Karambin et al. [21] from Iran, reported the *Entrobacter* (78.1%) as the most frequent bacterium contributed to neonatal septicemia. Although the bacterial agents responsible for neonatal sepsis are different depending on the geographical regions, it is not surprising to observe *Enterobacter* as the most common agent. In NICUs, the Gram-negative bacterium *Enterobacter cloacae* is notably responsible for nosocomial outbreaks [22]. Also, the gastrointestinal tract may serve as the source of these infections due to the fact that *Enterobacter* species colonize the neonatal microbiota at an early stage [22]. However, in another study by Fang et al. (23) from China, coagulase-negative staphylococci (CoNS) (36.52%) and *K. pneumoniae* were the most frequent pathogens contributed to neonatal early and late onset sepsis.

In the study period, all 59 (18.43%) isolates belonging to the *Enterobacterales* family were found to be MDR. In the next years, this high level of MDR *Enterobacterales* (MDRE) will be a serious threat. According to Folgori’s review, particularly in low and middle income countries (LMICs), poor outcome and high case fatality rates have been observed among neonates infected with MDRE [23]. Analysis of 30 studies consisting of 71,326 children indicated that the rate of MDR was 30% and 75% in Asia and Africa, respectively [24].

In this study, all *Enterobacterales* isolates were found to be ESBL-producer. Selective pressure caused by wide use of antibiotics in intensive care units (ICUs) may be mentioned as a reason for high percentage of ESBL producing isolates. According to the ERIC-PCR results, which showed a wide variety of isolates, it seems unlikely that this high prevalence of ESBL indicates the existence of an epidemic. Although, to prove it, there is a need for a more detailed investigation with a larger sample size and a more accurate typing method such as Multilocus Sequence Typing (MLST). Unfortunately, in Iran, due to the lack of appropriate monitoring programs for the prescription of antibiotics, their arbitrary consumption by patients without a doctor’s prescription, and their easy sale in pharmacies, the prevalence rate of ESBLs has increased. So far, despite the repeated reports of antibiotic resistance and ESBLs in the southwestern region of Iran [8, 14, 17], an integrated policy to control these global problems has not been implemented due to the lack of appropriate financial infrastructure.

In a study carried out by Ballot et al. 71% of all MDRE isolates were ESBL producer [25]. In another study, 67.3% of *Enterobacterales* isolates were ESBL producers as well [26]. In contrast with these studies, percentage of ESBL production in *Enterobacterales* isolates in the studies conducted by Manandhar et al. [27] and Charfi et al. [28] was low (25% and 16.5%), respectively. In a systemic review, prevalence of bloodstream infections (BSIs) with extended-spectrum beta-lactamase-producing *Enterobacterales* (ESBL-E) was 11% among neonates [29] while in our study was 18.4% (59/320). The treatment of infections in neonates caused by MDR *Enterobacterales* harboring ESBLs is challenging due to the limited availability of antibiotics. Colistin, fluoroquinolones, and tigecycline are not commonly used in this population due to their side effects [30]. The emergence of antibiotic-resistant pathogens is closely associated with the inappropriate and excessive use of broad-spectrum antibiotics. Additionally, outbreaks of infection in NICUs have been attributed to *Enterobacterales* pathogens that produce ESBL and carbapenemases, which are known to contribute to higher morbidity and mortality rates [31]. Also, neonates suffering from MDR infections tend to utilize a greater amount of resources for their treatment, as they typically experience more unfavorable clinical outcomes in comparison to patients with non-resistant infections, as viewed from a health economics standpoint [32, 33].

In this study the prevalence of ESBL genes was also assessed. The most frequency of ESBL genes belonged to *bla*_CTX−M−15_ (66.1%) that was in line with some previous studies [28, 34]. While in our study prevalence of *bla*_TEM_ gene was the lowest (13.6%), in the study done by Manandhar et al. percentage of this gene was the highest (53%). Additionally, percentage of *bla*_CTX−M_ and *bla*_SHV_ genes has been reported 26% and 15% respectively [27]. In the study performed by Chelliah et al. [26] *bla*_TEM_ gene in 22.4% of isolates was observed while *bla*_CTX−M_ and *bla*_SHV_ genes were not found. In the study done by Charfi et al. [28] 100% of *Enterobacterales* isolates harbored *bla*_CTX−M−15_ gene and most of strains were positive for *bla*_TEM_ (65.5%) and *bla*_SHV_ (78.2%) genes that is higher than our study. In Breurec’s study, *bla*_CTX−M−15_, *bla*_SHV_ and *bla*_TEM_ were very frequent (63.5%, 65.4% and 53.8% respectively) among third-generation cephalosporin resistant *Enterobacterales* isolates [35].

Production of AmpC was also evaluated in this study. Microbiologists face a challenge for detection of AmpC beta-lactamases owing to lack of standard guideline by CLSI to identify AmpC enzymes. The presence of AmpC-producing isolates creates a significant diagnostic hurdle due to the ineffectiveness of ESBL inhibitors, such as clavulanic acid, on AmpC enzymes. This interference complicates the identification of ESBL production [36]. Hence, the incorrect classification of these microorganisms as “non-ESBL-producing” leads to the misconception that they are not resistant to multiple drugs [36]. The potential for misleading results in phenotypic tests for AmpC presents a significant challenge to the specificity and sensitivity of these tests. This challenge, if not addressed, can have a detrimental effect on the accuracy of surveillance and hospital infection control measures [37]. The scarcity of accurate prevalence data of AmpC beta-lactamases can be attributed to the lack of standard method to detect these enzymes [38]. In present research 84.7% of isolates were AmpC positive. The prevalence of AmpC production in *Enterobacterales* isolates in neonatal septicemia has been reported from 1.1 to 23% [25, 26, 35, 39, 40]. Various methods used in studies can lead to different results.

Another aspect of this study was assessment of the frequency of AmpC genes in *Enterobacterales* isolates. Percentage of both *bla*_DHA_ and *bla*_EBC_ genes was 24% that followed by *bla*_MOX_ (4%) and *bla*_CIT_ (2%) genes. In a study by Breurec et al. [35], percentage of AmpC genes was 3.8% (*bla*_CIT_ gene in one *K. pneumoniae* isolate and *bla*_CMY2_ and *bla*_DHA_ in one *Enterobacter cloacae* were recognized). In the study performed by Husickova et al. [39], AmpC genes were discerned in 0.8% (*n* = 12/1526) of *Enterobacterales* isolates. EBC type, CIT type, DHA type and MOX type AmpC beta-lactamases were observed in 8 (66.6%), 2 (16.6%), 1 (8.3%), and 1 (8.3%) isolates respectively [39]. In another study by Jin et al. [41], a total of 37 *K. pneumoniae* isolates were examined for the presence of AmpC genes that only DHA type AmpC beta-lactamase in 28 (75.68%) isolates was observed. In a study by Roy et al. [42] from India, none of AmpC genes was found in *K. pneumoniae* and *E. coli* causing neonatal sepsis. In our study by comparison with two mentioned studies above, DHA type AmpC beta-lactamase in two (16.6%) of *K. pneumoniae* isolates was identified and three (25%) of isolates were positive for EBC type AmpC beta-lactamase. Furthermore, in one *E. coli* isolate DHA Type AmpC beta-lactamase and in another isolate CIT type and EBC type AmpC beta-lactamase were observed.

There was a high level of genetic diversity among *Enterobacterales* isolates using ERIC-PCR. In fact, the genetic diversity isolates illustrated non-clonal distribution of these isolates in NICU of two studied hospitals. Obtained result was in accordance with recent study carried out in Ahvaz and other studies in Iran as well, which displayed the genetic diversity in *Enterobacterales* family members such as *E. coli* and *K. pneumoniae* by ERIC-PCR technique [12, 43, 44]. In a previous study by Kundu et al. [45], ERIC-PCR emerges as a more reliable and effective typing tool when compared to matrix-assisted laser desorption ionization time-of-flight mass spectrometry (MALDI-TOF) for determining clonal relatedness in MDR *K. pneumoniae* clinical isolates. The *Enterobacterales* can be molecularly typed using ERIC-PCR, a method known for its speed, reliability, and cost-effectiveness [45]. The importance of using the ERIC-PCR in this study was to investigate the genetic relatedness of *Enterobacterales* causing neonatal sepsis and adopting strategies to control the infection caused by them. Although the findings of ERIC-PCR showed a great variety of species, but based on these findings, it seems necessary to use more accurate typing methods including multilocus sequence typing (MLST).

Based on the observed results, the significance of hygiene measures in NICUs is highlighted in our study [30]. These measures are essential for preventing the emergence and transmission of MDR bacteria [30, 31]. It is crucial to establish systematic surveillance systems and employ effective methods for detecting antimicrobial susceptibility as a future prospective. This will enhance antibiotic stewardship and ensure adherence to infection control measures, such as hand hygiene and surface cleaning [30, 31].

### Limitations

One of the most important limitations of the current study is the small sample size. Another limitation was the lack of sequencing of ESBL and AmpC genes to determine their variants. The lack of a more accurate technique in strain typing, such as MLST, was another limitation of the study. The lack of determine the genus and species of Gram-positive and non-*Enterobacterales* isolates was another limitation.

## Conclusions

This study verifies that there is a warning rise in MDRE conveying ESBL and AmpC beta-lactamase genes. It is essential to heighten antimicrobial stewardship efforts and infection control and prevention in neonatal units. In particular, there is an urgent need to discourage from the overuse of broad-spectrum antibiotics.

### Electronic supplementary material

Below is the link to the electronic supplementary material.

Supplementary Material 1

## Data Availability

The datasets used and/ or analyzed during the current study are available from the corresponding author on reasonable request.

## Abbreviations

AST: Antimicrobial susceptibility testing
EOS: Early-onset neonatal sepsis
ESBL: Extended-spectrum beta-lactamase
ERIC-PCR: Enterobacterial Repetitive Intergenic Consensus Polymerase Chain Reaction
LOS: Late-onset sepsis
MDRE: Multidrug-resistant *Enterobacterales*
