# Supplementary material for: High prevalence of multidrug-resistant Enterobacterales carrying extended-spectrum beta-lactamase and AmpC genes isolated from neonatal sepsis in Ahvaz, Iran
Source: BMC Microbiol. 2024 Apr 24;24:136. doi: 10.1186/s12866-024-03285-6 (PMC11040821; doi:10.1186/s12866-024-03285-6)
Supplement: Supplementary file 1 — Supplementary Material 1 [file 12866_2024_3285_MOESM1_ESM.docx]

Supplementary files 1. Characteristics of 59 *Enterobacterales* isolates.

|  |  | patients | | patients |  | Resistance genes | |
| --- | --- | --- | --- | --- | --- | --- | --- |
| ID  isolates | Bacterial isolate | Gender of the patient | Type of sepsis | Weight | Resistance pattern | Profile of ESBLs genes | Profile of AmpCs  genes |
| EB1 | *Enterobacter*  *spp.* | F | LOS | < 2000 | GEN, AMK, CHL, FOX, IMI, SAM ,TZP, SXT , CAZ, CTX | *bla*_CTX-M_,*bla*_CTX-M-15_ | *bla*_DHA_ |
| EB2 | *Enterobacter*  *spp.* | M | EOS | 2000-2500 | GEN, AMK, CHL, MEM, IMI, CIP,SAM , TZP, SXT,CAZ,CTX, AMP | *bla*_TEM_,*bla*_CTX-M-15_ | *bla*_DHA_ |
| EB3 | *Enterobacter*  *spp.* | F | EOS | < 2000 | GEN, CHL, MEM, FOX, AMK, IMI, CIP, SAM, TZP,SXT ,CAZ, CTX, AMP | *bla*_CTX-M-14_ | *bla*_EBC_ |
| EB4 | *Enterobacter*  *spp.* | M | EOS | 2000-2500 | GEN, AMK, CHL, MEM, FOX, CIP, SAM, SXT, CAZ, CTX | *bla*_TEM_, *bla*_SHV_ | *bla*_DHA_ |
| EB5 | *Enterobacter*  *spp.* | F | EOS | < 2000 | GEN, CHL, MEM, FOX, AMK, IMI, CIP, SAM, TZP,SXT ,CAZ, CTX, AMP | *bla*_CTX-M-15_ | *bla*_DHA_, *bla*_MOX_ |
| EB6 | *Enterobacter*  *spp.* | M | LOS | 2000-2500 | GEN, CHL, MEM, SAM, CAZ,CTX ,AMP | *bla*_CTX-M_, *bla*_CTX_-_M-14_, *bla*_CTX-M-15_ | NP |
| EB7 | *Enterobacter*  *spp.* | M | EOS | > 2500 | GEN, AMK, CHL, MEM, FOX, CIP, SAM, SXT, CAZ, CTX | *bla*_CTX-M-15_ | ND |
| EB8 | *Enterobacter*  *spp.* | F | EOS | < 2000 | GEN, CHL, MEM, FOX, AMK, IMI, CIP, SAM, TZP,SXT ,CAZ, CTX, AMP | *bla*_SHV_, *bla*_CTX_-_M-15_ | ND |
| EB9 | *Enterobacter*  *spp.* | M | EOS | 2000-2500 | GEN, AMK, CHL, IMI, CIP, SAM CAZ, CTX, AMP | *bla*_SHV_,*bla*_CTX -M_ | NP |
| EB10 | *Enterobacter*  *spp.* | M | EOS | > 2500 | GEN, AMK, CHL, FOX, IMI, MEM, CIP, SAM, SXT, CAZ, CTX | *bla*_SHV_ | *bla*_EBC_ |
| EB11 | *Enterobacter*  *spp.* | F | EOS | < 2000 | GEN, CHL, MEM, FOX, AMK, IMI, CIP, SAM, TZP,SXT ,CAZ, CTX, AMP | *bla*C_TX-M-14_, *bla*_CTX-M-15_ | ND |
| EB12 | *Enterobacter*  *spp.* | M | LOS | > 2500 | GEN, AMK, CHL, FOX , IMI, CIP, SAM , TZP, SXT, CAZ, CTX, AMP | *bla*_CTX-M_ | ND |
| EB13 | *Enterobacter*  *spp.* | M | EOS | 2000-2500 | GEN, AMK, CHL, FOX , IMI, CIP, SAM , TZP, SXT, CAZ, CTX, AMP | *bla*_SHV_,*bla*_CTX-M-15_ | ND |
| EB14 | *Enterobacter*  *spp.* | F | LOS | >2500 | GEN, CHL, MEM, FOX, AMK, IMI, CIP, SAM, TZP,SXT ,CAZ, CTX, AMP | *bla*_CTX-M-14_, *bla*_CTX-15_ | ND |
| EB15 | *Enterobacter*  *spp.* | M | EOS | < 2000 | GEN, AMK, CHL, FOX , IMI, CIP, SAM , TZP, SXT, CAZ, CTX, AMP | *bla*_SHV_ | ND |
| EB16 | *Enterobacter*  *spp.* | F | EOS | > 2500 | GEN, AMK, CHL, FOX, IMI, MEM, CIP, SAM, SXT, CAZ, CTX | *bla*_SHV_, *bla*_CTX-M_ | *bla*_EBC_ |
| EB17 | *Enterobacter*  *spp.* | M | LOS | < 2000 | GEN, AMK, MEM, FOX , CIP, SAM , TZP, SXT,CAZ, CTX, AMP | *bla*_CTX-M,_ bla_SHV,_bla_CTX-M-15_ | ND |
| EB18 | *Enterobacter*  *spp.* | F | EOS | 2000-2500 | GEN, CHL, MEM, FOX, AMK, IMI, CIP, SAM, TZP,SXT ,CAZ, CTX, AMP | *bla*_CTX-M ,_ *bla*_CTX-M-14_ | *bla*_DHA_ |
| EB19 | *Enterobacter*  *spp.* | M | EOS | < 2000 | GEN, AMK, MEM, FOX , CIP, SAM , TZP, SXT,CAZ, CTX, AMP | *bla*_CTX-M_ | ND |
| EB20 | *Enterobacter*  *spp.* | M | EOS | > 2500 | GEN, AMK, CHL, MEM, FOX, IMI, TZP, SXT,CAZ,CTX, AMP | *bla*_CTX-M-15_ | ND |
| EB21 | *Enterobacter*  *spp.* | F | EOS | > 2500 | GEN, AMK, CHL, FOX, IMI, MEM, CIP, SAM, SXT, CAZ, CTX | *bla*_CTX-M-14_, *bla*_CTX-M-15_ | ND |
| EB22 | *Enterobacter*  *spp.* | M | LOS | 2000-  2500 | GEN, AMK, CHL, MEM, FOX, CIP, SAM, SXT, CAZ, CTX | *bla*_CTX-M_ | ND |
| EB23 | *Enterobacter*  *spp.* | F | EOS | < 2000 | GEN, AMK, CHL, IMI, SAM, TZP, SXT,CAZ, CTX, AMP | *bla*_CTX-M_ ,bla_CTX-M-15_ | NP |
| EB24 | *Enterobacter*  *spp.* | M | LOS | 2000-  2500 | GEN, CHL, MEM, FOX, AMK, IMI, CIP, SAM, TZP,SXT ,CAZ, CTX, AMP | *bla*_CTX-M-14_,bla_CTX-M-15_ | *bla*_MOX_, *bla*_DHA_ |
| EB25 | *Enterobacter*  *spp.* | F | EOS | <2000 | GEN, CHL, MEM, FOX, AMK, IMI, CIP, SAM, TZP,SXT ,CAZ, CTX, AMP | *bla*_CTX-M-14_ | ND |
| EB26 | *Enterobacter*  *spp.* | M | EOS | < 2000 | GEN, AMK, CHL, FOX, IMI, MEM, CIP, SAM, SXT, CAZ, CTX | *bla*_CTX-M-15_ | ND |
| EB27 | *Enterobacter*  *spp.* | M | EOS | 2000-2500 | GEN, AMK, CHL, FOX , IMI, CIP, SAM , TZP, SXT, CAZ, CTX, AMP | *bla*_CTX-M,_ bla_SHV,_bla_CTX-M-15_ | ND |
| EB28 | *Enterobacter*  *spp.* | F | LOS | < 2000 | GEN, CHL, MEM, FOX, IMI, SAM, SXT, CAZ, CTX | *bla*_CTX-M-14_ | *bla*_DHA_ |
| EB29 | *Enterobacter*  *spp.* | M | EOS | > 2500 | GEN, CHL, MEM, FOX, AMK, IMI, CIP, SAM, TZP,SXT ,CAZ, CTX, AMP | *bla*_TEM,_*bla*_CTX-M-15_ | *bla*_EBC_ |
| EB30 | *Enterobacter*  *spp.* | F | EOS | < 2000 | GEN, AMK, CHL, MEM, IMI, CIP, SAM, SXT , CAZ, CTX | *bla*_CTX-M-14_ | NP |
| EB31 | *Enterobacter*  *spp.* | M | EOS | < 2000 | GEN, CHL, MEM, FOX, AMK, IMI, CIP, SAM, TZP,SXT ,CAZ, CTX, AMP | *bla*_TEM,_ *bla*_SHV_ | *bla*_EBC_ |
| EB32 | *Enterobacter*  *spp.* | M | LOS | < 2000 | GEN, AMK, MEM, IMI, CIP, SAM , TZP, SXT, CAZ, CTX | *bla*_CTX-M ,_ *bla*_CTX-M-15_ | NP |
| EB33 | *Enterobacter*  *spp.* | F | EOS | 2000-2500 | GEN, AMK, CHL, MEM, IMI, CIP, SAM, SXT , CAZ, CTX | *bla*_CTX-M_ , *bla*_CTX-M-15_ | NP |
| EB34 | *Enterobacter*  *spp.* | F | EOS | < 2000 | GEN, CHL, MEM, FOX, AMK, IMI, CIP, SAM, TZP,SXT ,CAZ, CTX, AMP | *bla*_CTX-M,_bla_CTX-15_ | *bla*_EBC_ |
| EB35 | *Enterobacter*  *spp.* | M | LOS | 2000-2500 | GEN, AMK, MEM, IMI, CIP, SAM , TZP, SXT, CAZ, CTX | *bla*_CTX-M-15_ | NP |
| EB36 | *Enterobacter*  *spp.* | F | EOS | < 2000 | GEN, AMK, MEM, IMI, CIP, SAM , TZP, SXT, CAZ, CTX | *bla*_CTX-M,_ *bla*_CTX-M-15_ | NP |
| EB37 | *Enterobacter*  *spp.* | M | LOS | > 2500 | GEN, CHL, MEM, FOX, AMK, IMI, CIP, SAM, TZP,SXT ,CAZ, CTX, AMP | *bla*_CTX-M-14,_bla_CTX-M-15_ | *bla*_EBC_ |
| EB38 | *Enterobacter*  *spp.* | M | EOS | < 2000 | GEN, AMK, MEM, IMI,CIP, SAM, SXT, CAZ, CTX | *bla*_CTX-M_ | *bla*_EBC_, *bla*_DHA_ |
| EB39 | *Enterobacter*  *spp.* | F | EOS | 2000-2500 | GEN, AMK, CHL, MEM, IMI, CIP,SAM , TZP, SXT,CAZ,CTX, AMP | *bla*_CTX-M,_*bla*_CTX-M-15_ | NP |
| EB40 | *Enterobacter*  *spp.* | M | LOS | > 2500 | GEN, CHL, MEM, FOX, AMK, IMI, CIP, SAM, TZP,SXT ,CAZ, CTX, AMP | *bla*_CTX-M-14_ | *bla*_DHA_ |
| EB41 | *Enterobacter*  *spp.* | F | EOS | > 2500 | GEN, CHL, MEM, FOX, AMK, IMI, CIP, SAM, TZP,SXT ,CAZ, CTX, AMP | *bla*_CTX-M-14,_ *bla*_CTX-M_ | ND |
| EB42 | *K. pneumoniae* | M | EOS | > 2500 | GEN, CHL, MEM, AMK, FOX, CIP, SAM, TZP, SXT, CAZ, CTX, AMP | *bla*_CTX-M-15_ | ND |
| EB43 | *K. pneumoniae* | F | EOS | > 2500 | GEN, CHL, MEM, AMK, FOX, CIP, SAM, TZP, SXT, CAZ, CTX, AMP | *bla*_SHV,_ *_bla_*_CTX-M15_ | *bla*_EBC_ |
| EB44 | *K. pneumoniae* | F | EOS | > 2500 | GEN, CHL, MEM, FOX, AMK, IMI, CIP, SAM, TZP,SXT ,CAZ, CTX, AMP | *bla*_SHV,_*bla*_CTX-M-14_*,bla*_CTX-M-15_ | *bla*_DHA_ |
| EB45 | *K. pneumoniae* | F | LOS | < 2000 | GEN, AMK, CHL, MEM, FOX , IMI, SAM , TZP , CIP , CAZ, CTX , AMP | *bla*_CTX-M-14_ | ND |
| EB46 | *K. pneumoniae* | M | EOS | < 2000 | GEN, AMK, CHL, MEM, IMI, CIP, SAM, SXT , CAZ, CTX | *bla*_CTX-M,_ *bla*_CTX-M-15_ | *bla*_EBC_ |
| EB47 | *K. pneumoniae* | F | EOS | > 2500 | GEN, AMK, CHL, MEM, FOX , IMI, SAM , TZP , CIP , CAZ, CTX , AMP | *bla*_CTX-M,_ *bla*_SHV,_ *bla*_CTX-M-14_ | ND |
| EB48 | *K. pneumoniae* | M | LOS | < 2000 | GEN, AMK, CHL, MEM, IMI, CIP,SAM , TZP, SXT,CAZ,CTX, AMP | _M,_*bla*_SHV,_*bla*_CTX-M-15_ | *bla*_DHA_ |
| EB49 | *K. pneumoniae* | F | EOS | > 2500 | GEN, CHL, MEM, FOX, AMK, IMI, CIP, SAM, TZP,SXT ,CAZ, CTX, AMP | *bla*_CTX-M,_*bla*_TEM,_*bla*_SHV,_*bla*_CTX-M-15_ | *bla*_EBC_ |
| EB50 | *K. pneumoniae* | M | EOS | < 2000 | GEN, CHL, MEM, FOX, AMK, IMI, CIP, SAM, TZP,SXT ,CAZ, CTX, AMP | *bla*_CTX-M ,_ *bla*_CTX-M-15_ | ND |
| EB51 | *K. pneumoniae* | M | LOS | 2000-2500 | GEN, AMK, CHL, MEM, FOX , IMI, SAM , TZP, SXT, CAZ, CTX, AMP | *bla*_SHV,_ *bla*_CTX-M-15_ | ND |
| EB52 | *K. pneumoniae* | F | LOS | > 2500 | GEN, CHL, MEM, FOX, AMK, IMI, CIP, SAM, TZP,SXT ,CAZ, CTX, AMP | *bla*_SHV,_*bla*_TEM,_*bla*_CTX-M-15_ | ND |
| EB53 | *K. pneumoniae* | M | EOS | < 2000 | GEN, AMK, CHL, MEM, FOX , IMI, SAM , TZP, SXT, CAZ, CTX, AMP | *bla*_CTX-M-15,_ *bla*_CTX-M_ | ND |
| EB54 | *K. pneumoniae* | F | LOS | > 2500 | CHL, AMK, FOX, IMI, TZP, SXT, CAZ, CTX | *bla*_CTX-M-15_ | ND |
| EB55 | *K. pneumoniae* | F | EOS | < 2000 | CHL, AMK, FOX, IMI, TZP, SXT, CAZ, CTX | *bla*_CTX-M-15,_ *bla*_CTX-M-14_ | ND |
| EB56 | *K. pneumoniae* | M | EOS | < 2000 | GEN, AMK, CHL, MEM, FOX , IMI, SAM , TZP, SXT, CAZ, CTX, AMP | *bla*_TEM,_ *bla*_CTX-M_ | ND |
| EB57 | *E. coli* | F | LOS | > 2500 | GEN, AMK, CHL, MEM, FOX , IMI, SAM , TZP, SXT, CAZ, CTX, AMP | *bla*_TEM,_ *bla* _CTX-M-14,_*bla* _CTX-M-15_ | *bla*_CIT_, *bla*_EBC_, |
| EB58 | *E. coli* | M | EOS | < 2000 | GEN, AMK, CHL, MEM, FOX , IMI, SAM , TZP, SXT, CAZ, CTX, AMP | *bla*_CTX-M-15_ | ND |
| EB59 | *E. coli* | M | EOS | 2000-2500 | GEN, CHL, MEM, FOX, AMK, IMI, CIP, SAM, TZP,SXT ,CAZ, CTX, AMP | *bla*_CTX-M-15 ,_ *bla*_CTX-M_ | ND |

F: female, M: male, EOS: early-onset sepsis, LOS: late-onset sepsis, NP: not performed, ND: not detected, GEN: gentamicin, CHL: chloramphenicol, MEM: meropenem, FOX: cefoxitin, AMK: amikacin, IMI: imipenem, CIP: ciprofloxacin, SAM: ampicillin-sulbactam, TZP: piperacillin-tazobactam, SXT: cotrimoxazole, CAZ: ceftazidime, CTX: cefotaxime, AMP: ampicillin
